# Conversion Surgery for Alpha-Fetoprotein–Producing Esophagogastric Junction Cancer with Multiple Liver Metastases and Portal Vein Tumor Thrombus after Nivolumab Combination Chemotherapy: A Case Report and Literature Review

**DOI:** 10.70352/scrj.cr.25-0808

**Published:** 2026-02-26

**Authors:** Masaaki Akai, Shoji Takagi, Tomohiro Toji, Yoshifumi Mitani, Mikoto Shimabara, Yuta Nobunaga, Toshihisa Matsumura, Masafumi Inoue

**Affiliations:** 1 Department of Gastroenterological Surgery, Okayama Red Cross Hospital, Okayama, Okayama, Japan; 2 Department of Pathology, Okayama Red Cross Hospital, Okayama, Okayama, Japan; 3 Department of Gastroenterology, Okayama Red Cross Hospital, Okayama, Okayama, Japan

**Keywords:** esophagogastric junction cancer, alpha-fetoprotein–producing gastric cancer, nivolumab, conversion surgery

## Abstract

**INTRODUCTION:**

Alpha-fetoprotein–producing gastric cancer (AFPGC) is a rare subtype of gastric cancer associated with poor prognosis due to early liver metastasis. There have been limited reports on conversion surgery following nivolumab combination chemotherapy in cases of AFPGC with liver metastasis. Here, we present a case of alpha-fetoprotein–producing esophagogastric junction cancer (AFP-EGJC) successfully treated with this treatment.

**CASE PRESENTATION:**

The patient was a 31-year-old man. After liver tumors were incidentally detected, he was diagnosed with esophagogastric junction cancer (cT3N1M1) with multiple liver metastases and portal vein tumor thrombus. Combination therapy with S-1 (tegafur/gimeracil/oteracil), oxaliplatin, and nivolumab was initiated. The response was remarkable, with the rapid disappearance of liver metastases. Serum AFP was also abnormally high at 691.9 ng/mL, but it quickly normalized after the start of treatment. Fifteen months later, the patient was diagnosed with progressive disease, and the regimen was switched to nab-paclitaxel and ramucirumab therapy. However, there was no recurrence of liver metastases, and only the primary tumor was poorly controlled. Therefore, a laparoscopic proximal gastrectomy was performed 22 months after the initial treatment. The postoperative diagnosis was ypT2 (MP) N0M0, ypStage IB. The patient remains alive and recurrence-free 12 months after surgery, without adjuvant chemotherapy.

**CONCLUSIONS:**

Conversion surgery after nivolumab combination chemotherapy may be feasible in selected patients with AFP-EGJC with liver metastasis.

## Abbreviations

AFP
: alpha-fetoprotein
AFP-EGJC
: alpha-fetoprotein–producing esophagogastric junction cancer
AFPGC
: alpha-fetoprotein–producing gastric cancer
CA19-9
: carbohydrate antigen 19-9
CEA
: carcinoembryonic antigen
CPS
: combined positive score
CR
: complete response
CS
: conversion surgery
GC
: gastric cancer
ICI
: immune checkpoint inhibitor
Nivo
: nivolumab
PD-L1
: programmed death-ligand 1
SOX
: S-1 and oxaliplatin

## INTRODUCTION

As GC is the fourth leading cause of cancer deaths worldwide and the fifth most frequently diagnosed cancer, it presents a significant global health problem.^1)^ The 5-year overall survival rate of patients with Stage IV GC is only 7%.^2)^ In recent years, immunotherapy, particularly ICIs, has garnered attention as a promising treatment strategy for GC. Recent clinical trials, the CheckMate649^3)^ and ATTRACTION-4^4)^ studies, demonstrated the efficacy of nivolumab-based combination chemotherapy as a first-line regimen for advanced or metastatic HER2-negative gastric adenocarcinoma. In Japan, the GC guidelines have recommended this regimen since November 2021.^5)^ CS has also attracted attention as a treatment for Stage IV GC, and some reports indicate that R0 resection can lead to longer survival.^6)^

AFP is an oncofetal protein.^7)^ Elevated serum AFP levels in adults are used as a clinical biomarker for hepatocellular carcinoma or yolk sac tumors.^8,9)^ AFPGC is a rare type of GC; the reported incidence of GC is 1.3%–15%.^10)^ AFPGC has a poor prognosis and is characterized by higher rates of venous invasion, lymphatic invasion, and metachronous or synchronous liver metastases than other GCs.^11–13)^

There have been limited reports on CS following nivolumab combination chemotherapy in cases of AFPGC with liver metastasis. Here, we present a patient with AFP-EGJC who underwent this treatment.

## CASE PRESENTATION

**Fig. 1:**
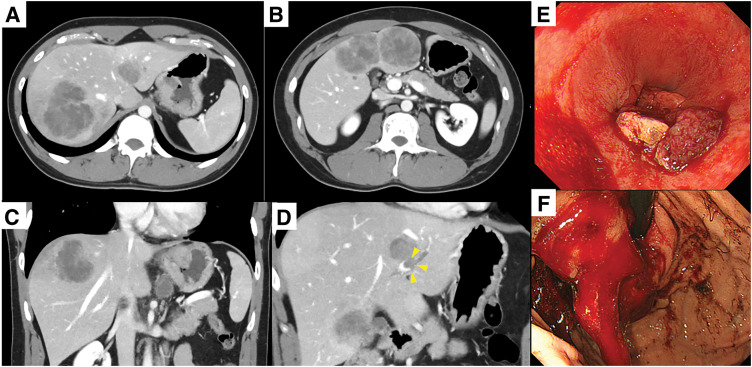
Contrast-enhanced CT and upper gastrointestinal endoscopy image before therapy. (**A**, **B**) Multiple liver metastases with a maximum diameter of 7.5 cm. (**C**) Primary esophagogastric junction cancer and lymph node metastasis. (**D**) Portal vein tumor thrombus (arrowheads). (**E**, **F**) Advanced esophagogastric junction cancer (macroscopic type 2; esophageal invasion: 1.5 cm; gastric invasion: 4 cm).

A 31-year-old man with no significant medical history was incidentally noted to have liver tumors. Upper gastrointestinal endoscopy revealed advanced esophagogastric junction cancer (macroscopic type 2; esophageal invasion: 1.5 cm; gastric invasion: 4 cm) (**Fig. 1**). A biopsy confirmed poorly differentiated adenocarcinoma that was HER2-negative and AFP-positive. The PD-L1 CPS of this tumor was more than 5. Blood tests revealed a CEA level of 8.1 ng/mL, CA19-9 level of 58 U/mL, and an AFP level of 691.9 ng/mL. Contrast-enhanced CT revealed multiple liver metastases in both lobes with a portal vein tumor thrombus. The primary tumor appeared as a contrast-enhancing thickening of the gastric wall without serosal invasion. One enlarged lymph node was detected along the lesser curvature of the stomach. Based on these findings, the patient was diagnosed with AFP-EGJC, classified as clinical Stage IVB (cT3N1M1). Diagnostic laparoscopy was not performed at the initial presentation because multiple liver metastases were evident, and priority was given to prompt initiation of systemic therapy.

**Fig. 2:**
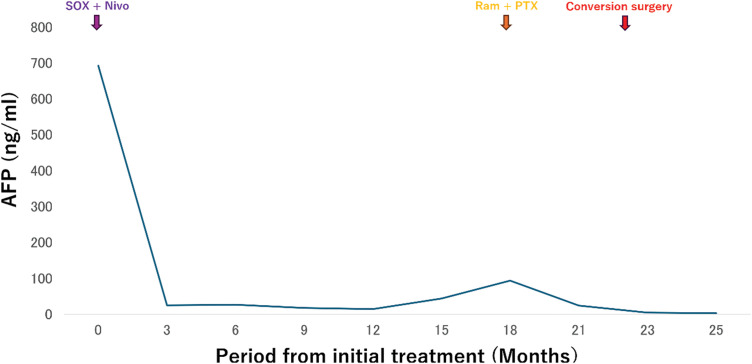
Therapeutic course and serum level of AFP. AFP, alpha-fetoprotein; Ram + PTX, ramucirumab and nab-paclitaxel; SOX + Nivo, S-1, oxaliplatin, and nivolumab

**Fig. 3:**
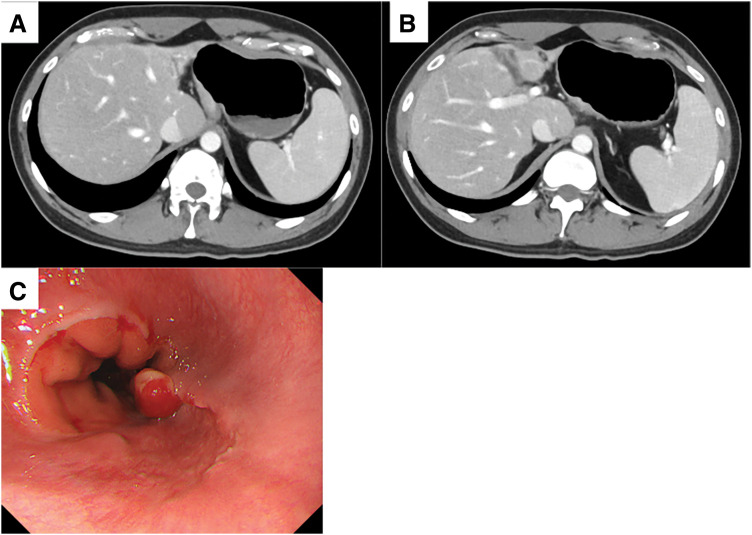
Contrast-enhanced CT and upper gastrointestinal endoscopy image before conversion surgery. (**A**, **B**) Multiple liver metastases and the portal vein tumor thrombus disappeared, and gastric wall thickening and lymph node swelling also disappeared. (**C**) Upper gastrointestinal endoscopy revealed areas of scarring due to treatment and elevated lesions suggestive of residual cancer.

**Fig. 4:**
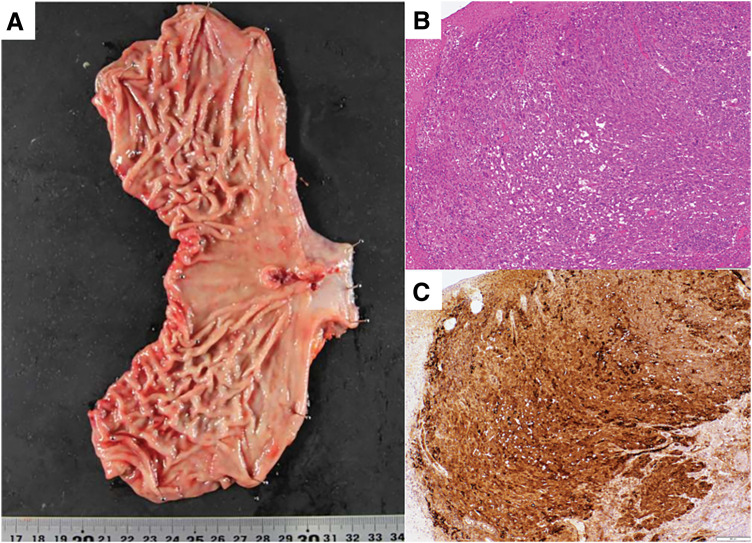
Resected specimen. (**A**) Macroscopic picture. (**B**) HE-stained image at ×40 magnification. Scale bar = 200 μm. (**C**) AFP immunostaining at ×40 magnification. Scale bar = 200 μm. AFP, alpha-fetoprotein; HE, hematoxylin and eosin

The patient was started on a regimen of S-1, oxaliplatin, and nivolumab (SOX + Nivo) as first-line chemotherapy. The tumor demonstrated a remarkable response, and a CT scan taken 3 months after treatment initiation showed a significant reduction in liver metastases. One year after starting therapy, the liver metastases achieved CR, with no detectable lesions on CT. Serum AFP levels also declined significantly, reaching 14 ng/mL after 1 year (**Figs. 2** and **3**). However, 15 months after treatment initiation, serum AFP levels increased, and progression was observed only in the primary lesion, with no recurrence of liver metastases. Second-line chemotherapy with nab-paclitaxel and ramucirumab was initiated. After 1 course, the tumor showed slight shrinkage, and curative resection via a transhiatal approach was considered feasible. Twenty-two months after initial treatment, the patient underwent laparoscopic proximal gastrectomy as CS. At the time of CS, the abdominal cavity was carefully inspected at the beginning of the procedure, and the absence of macroscopic peritoneal dissemination was confirmed before proceeding with tumor resection. The postoperative course was uneventful, and the patient was discharged on POD 9. Pathological examination confirmed ypStage IB (ypT2N0M0). The pathological response to chemotherapy was classified as Grade 1b, and immunohistochemistry showed diffuse AFP positivity (**Fig. 4**). After surgery, serum AFP levels returned to within the normal range. The patient remains alive and disease-free without adjuvant chemotherapy 12 months postoperatively.

## DISCUSSION

AFPGC is known to have a poor prognosis.^11–13)^ Esophagogastric junction cancer has a worse prognosis than GC.^14)^ Therefore, AFP-EGJC is considered to have an especially unfavorable prognosis. In this case, SOX + Nivo therapy was remarkably effective against an aggressive AFP-EGJC with multiple liver metastases, ultimately enabling curative resection via CS.

Numerous studies have reported the poor prognosis of AFPGC. According to a literature review by Ota et al.,^15)^ the median overall survival time was 14–72 months, and the 5-year survival rate was 8.3%–66%. Another review focusing on AFPGC in Japan reported that while high serum AFP levels were commonly seen in cases with liver metastases, AFP itself was not an independent prognostic factor.^16)^ Furthermore, it has been suggested that elevated AFP levels may correlate with reduced effectiveness of ICIs, as AFP may suppress antitumor immunity.^17)^

In the present case, although the initial AFP level was markedly elevated, it decreased dramatically in response to therapy. This suggests that a rapid decline in serum AFP levels may serve as an indicator of strong treatment response.

While several reports have demonstrated the effectiveness of chemotherapy in AFPGC,^18,19)^ reports on the efficacy of ICIs remain limited.^15,20,21)^ Most previously reported cases involving ICIs were in the context of recurrent tumors or gastric remnant cancer. To our knowledge, no prior cases have reported CS after first-line ICI-based therapy for AFP-EGJC.

When discussing CS for Stage IV GC, the Yoshida classification^22)^ provides a useful framework for patient selection. According to this classification, Stage IV disease is categorized based on resectability and response to systemic therapy, and CS is mainly considered for patients classified as Category 2, in whom initially unresectable disease becomes marginally resectable after chemotherapy.

In the present case, the patient would be classified as Category 2, as multiple liver metastases initially rendered the disease unresectable, but CR of the metastatic lesions and tumor shrinkage following systemic therapy enabled complete resection. This case supports the concept that CS may be considered in carefully selected patients; however, its indication, optimal timing, and long-term benefit remain to be clarified.

GC with liver metastasis is generally associated with poor prognosis, and systemic chemotherapy is considered the standard treatment. However, several studies have suggested that selected patients with liver-limited metastases may benefit from surgical intervention when a favorable response to chemotherapy is achieved.^23,24)^ In recent years, ICI-based combination chemotherapy has improved response rates in advanced GC, potentially increasing opportunities for CS in carefully selected patients. Although ICIs have improved response rates in advanced GC, their impact on the indications and long-term outcomes of CS remains to be fully elucidated.

In the present case, distant metastases were confined to the liver and achieved CR with nivolumab-based chemotherapy, which enabled curative resection. Nevertheless, such outcomes are exceptional, and the optimal indications, treatment strategies, and long-term prognosis for GC with liver metastasis remain to be clarified through further clinical experience and prospective studies.

The optimal timing of CS in such cases remains controversial. In our patient, CS was initially considered when the first-line SOX + Nivo therapy had achieved its maximum effect, but surgery was postponed due to a lack of consent. Several months later, progression of the primary tumor was observed, raising concern that further delay might lead to a loss of operability. Although the liver metastases remained in CR, the possibility of occult regrowth could not be completely excluded. If the liver lesions had relapsed, CS would have been limited to a debulking procedure, potentially compromising prognosis. After the initiation of second-line nab-paclitaxel plus ramucirumab, the primary tumor showed a favorable response and decreased in size, enabling resection via a transhiatal approach alone. At the same time, the liver metastases continued to show a sustained CR without progression. Based on these findings and the risk of further progression of the primary tumor, we judged that proceeding with CS at this early time point would maximize the chance of curative resection. Nevertheless, the optimal duration and sequencing of systemic therapy before CS remain controversial and require further investigation.

Pathological findings after CS revealed that there was no metastasis, even in the lymph nodes that had been enlarged before treatment. Based on the achievement of complete radical resection at CS, postoperative chemotherapy was discontinued. The patient remains alive and disease-free without adjuvant chemotherapy 12 months postoperatively. Although this favorable outcome was observed in a single case, further accumulation of cases is required to clarify appropriate patient selection and the optimal timing of CS.

## CONCLUSIONS

We presented a case of AFP-EGJC with multiple liver metastases that was successfully managed with first-line nivolumab-based combination chemotherapy followed by CS. This treatment strategy may be feasible in selected patients; however, further accumulation of similar cases is required.

## DECLARATIONS

### Availability of data and materials

All data generated or analyzed during this study are included in this published article.

## References

[1] Ilic M, Ilic I. Epidemiology of stomach cancer. World J Gastroenterol 2022; 28: 1187–203. 35431510 10.3748/wjg.v28.i12.1187 PMC8968487

[2] American Cancer Society. Stomach cancer early detection, diagnosis, and staging. 2025.

[3] Janjigian YY, Shitara K, Moehler M, et al. First-line nivolumab plus chemotherapy versus chemotherapy alone for advanced gastric, gastro-oesophageal junction, and oesophageal adenocarcinoma (CheckMate 649): a randomised, open-label, phase 3 trial. Lancet 2021; 398: 27–40. 34102137 10.1016/S0140-6736(21)00797-2 PMC8436782

[4] Kang YK, Chen LT, Ryu MH, et al. Nivolumab plus chemotherapy versus placebo plus chemotherapy in patients with HER2-negative, untreated, unresectable advanced or recurrent gastric or gastro-oesophageal junction cancer (ATTRACTION-4): a randomised, multicentre, double-blind, placebo-controlled, phase 3 trial. Lancet Oncol 2022; 23: 234–47. 35030335 10.1016/S1470-2045(21)00692-6

[5] Japanese Gastric Cancer Association. Japanese Gastric Cancer Treatment Guidelines 2021 (6th edition). Gastric Cancer 2023; 26: 1–25. 36342574 10.1007/s10120-022-01331-8 PMC9813208

[6] Takeno A, Motoori M, Kishi K, et al. Prognostic factors of conversion surgery for stage IV gastric cancer: a multi-institutional retrospective analysis. Ann Gastroenterol Surg 2024; 8: 431–42. 38707233 10.1002/ags3.12778 PMC11066490

[7] Soltani K. Alpha-fetoprotein: a review. J Invest Dermatol 1979; 72: 211–3. 88486 10.1111/1523-1747.ep12530749

[8] Tatarinov IuS. Detection of embryo-specific alpha-globulin in the blood serum of a patient with primary liver cancer (in Russian). Vopr Med Khim 1964; 10: 90–1. 14207501

[9] Norgaard-Pedersen B, Albrechtsen R, Teilum G. Serum alpha-foetoprotein as a marker for endodermal sinus tumour (yolk sac tumour) or a vitelline component of ‘teratocarcinoma’. Acta Pathol Microbiol Scand A 1975; 83: 573–89. 52995 10.1111/j.1699-0463.1975.tb01385.x

[10] Chun H, Kwon SJ. Clinicopathological characteristics of alpha-fetoprotein-producing gastric cancer. J Gastric Cancer 2011; 11: 23–30. 22076198 10.5230/jgc.2011.11.1.23 PMC3204474

[11] Chang YC, Nagasue N, Kohno H, et al. Clinicopathologic features and long-term results of alpha-fetoprotein-producing gastric cancer. Am J Gastroenterol 1990; 85: 1480–5. 1700600

[12] Chang YC, Nagasue N, Abe S, et al. Comparison between the clinicopathologic features of AFP-positive and AFP-negative gastric cancers. Am J Gastroenterol 1992; 87: 321–5. 1371637

[13] Kono K, Amemiya H, Sekikawa T, et al. Clinicopathologic features of gastric cancers producing alpha-fetoprotein. Dig Surg 2002; 19: 359–65; discussion 65. 12435906 10.1159/000065838

[14] Yanagimoto Y, Kurokawa Y, Doki Y, et al. Surgical and perioperative treatment strategy for resectable esophagogastric junction cancer. Jpn J Clin Oncol 2022; 52: 417–24. 35246684 10.1093/jjco/hyac019

[15] Ota T, Sakashita K, Sawada R, et al. Long-term survival with nivolumab followed by irinotecan after total gastrectomy in alpha-fetoprotein-producing gastric cancer: a case report and review of the literature. Surg Case Rep 2023; 9: 71. 37150760 10.1186/s40792-023-01653-4 PMC10164665

[16] Nanba N, Oba M, Kikuchi Y, et al. Prognosis of alpha-fetoprotein-produced gastric cancer in the japanese population: a systematic review of case reports. Toho J Med 2022; 8: 138–43.

[17] Zhang J, Wang L, Zhang S, et al. Alpha-fetoprotein predicts the treatment efficacy of immune checkpoint inhibitors for gastric cancer patients. BMC Cancer 2024; 24: 266. 38408930 10.1186/s12885-024-11999-z PMC10895833

[18] Furukawa S, Kobayashi T, Shiono S, et al. Metachronous liver metastasis from alpha-fetoprotein-producing gastric cancer successfully treated with capecitabine/oxaliplatin combination chemotherapy. Case Rep Surg 2022; 2022: 2700394. 36051651 10.1155/2022/2700394 PMC9427307

[19] Kamiimabeppu D, Wakatsuki T, Takahari D, et al. Treatment efficacy of ramucirumab-containing chemotherapy in patients with alpha-fetoprotein producing gastric cancer. Int J Clin Oncol 2023; 28: 121–9. 36409433 10.1007/s10147-022-02263-0

[20] Ono HA, Suwa H, Minami Y, et al. A case of juvenile AFP-produsing gastric cancer with virchow lymph node metastasis achieved long-term survival with muiltimodal therapy (in Japanese). Gan To Kagaku Ryoho 2023; 50: 1892–4. 38303243

[21] Katsumata K, Morimoto Y, Aoyama J, et al. Conversion surgery for gastric remnant cancer with liver metastasis after nivolumab combination chemotherapy achieving pathological complete response: a case report and literature review. Surg Case Rep 2024; 10: 107. 38691201 10.1186/s40792-024-01905-x PMC11063010

[22] Yoshida K, Yamaguchi K, Okumura N, et al. Is conversion therapy possible in stage IV gastric cancer: the proposal of new biological categories of classification. Gastric Cancer 2016; 19: 329–38. 26643880 10.1007/s10120-015-0575-z PMC4824831

[23] Takahashi K, Terashima M, Notsu A, et al. Surgical treatment for liver metastasis from gastric cancer: a systematic review and meta-analysis of long-term outcomes and prognostic factors. Eur J Surg Oncol 2024; 50: 108582. 39126987 10.1016/j.ejso.2024.108582

[24] Arigami T, Matsushita D, Okubo K, et al. Indication and prognostic significance of conversion surgery in patients with liver metastasis from gastric cancer. Oncology 2020; 98: 273–9. 32062663 10.1159/000505555

